# Traditional Disease Risk Factors Outperform Epigenetic Clocks as Predictors of Non‐Communicable Disease Morbidity in a Middle‐Aged Cohort

**DOI:** 10.1111/acel.70626

**Published:** 2026-07-09

**Authors:** Daria Kostiniuk, Flóra Székely, Leo‐Pekka Lyytikäinen, Jo Ciantar, Sonja Rajić, Pashupati P. Mishra, Terho Lehtimäki, Katja Pahkala, Suvi Rovio, Juha Mykkänen, Olli Raitakari, Emma Raitoharju, Saara Marttila

**Affiliations:** ^1^ Molecular Epidemiology (MOLE), Faculty of Medicine and Health Technology Tampere University Tampere Finland; ^2^ Institute of Genomic Medicine and Rare Disorders Semmelweis University Budapest Hungary; ^3^ Department of Clinical Chemistry, Faculty of Medicine and Health Technology Tampere University Tampere Finland; ^4^ Finnish Cardiovascular Research Center Tampere, Faculty of Medicine and Health Technology Tampere University Tampere Finland; ^5^ Fimlab Laboratories Tampere Finland; ^6^ Research Centre of Applied and Preventive Cardiovascular Medicine University of Turku Turku Finland; ^7^ Centre for Population Health Research University of Turku and Turku University Hospital Turku Finland; ^8^ Paavo Nurmi Centre, Unit for Health and Physical Activity University of Turku Turku Finland; ^9^ Department of Public Health University of Turku and Turku University Hospital Turku Finland; ^10^ Department of Clinical Physiology and Nuclear Medicine Turku University Hospital Turku Finland; ^11^ Tampere University Hospital, Wellbeing Services County of Pirkanmaa Tampere Finland; ^12^ Gerontology Research Center Tampere University Tampere Finland

**Keywords:** ageing, biological age, biomarker of age, epigenetic age, epigenetic clock

## Abstract

DNA methylation‐based epigenetic clocks have been highlighted as promising biomarkers of ageing, and they have been shown to robustly predict morbidity and mortality. However, current literature is lacking a formal analysis of the increased prediction accuracy, or the added value, of the epigenetic clocks over traditional risk factors, such as body composition, smoking, or alcohol consumption, in predicting common chronic diseases. Here, we have compared the most commonly used epigenetic clocks and traditional risk factors as predictors of incidence of ageing‐associated non‐communicable chronic disease in a 7‐to‐9‐year follow‐up in a middle‐aged population cohort (*n* = 1108, aged 34 to 49 years at baseline). In our cohort, a statistical model consisting of a combination of traditional risk factors (age, sex, smoking, alcohol consumption, WHR, BMI) outperforms any model including an epigenetic clock. The added value of epigenetic clock measurements over simple and affordable traditional risk factors should be clearly established if epigenetic clocks are to be used in clinical settings or as tools of personal health monitoring.

## Graphical abstract

A statistical model consisting of a combination of traditional risk factors (age, sex, smoking, alcohol consumption, WHR, BMI) outperforms any model including an epigenetic clock in predicting incidence of common chronic diseases (cardiometabolic diseases, hypertension, cancer, steatotic liver disease) during a 7‐to‐9‐year follow‐up in middle‐aged individuals.

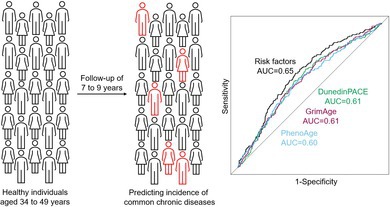

The concept of biological age aims to capture the amount of ageing‐associated damage that has accumulated over time, and biomarkers of ageing are measures of this biological age. By definition, biomarkers of ageing should be better predictors of future health outcomes than mere chronological age (Baker and Sprott 1988; Moqri et al. 2023). DNA methylation‐based epigenetic clocks have been highlighted as promising biomarkers of ageing (Moqri et al. 2023; Duan et al. 2022). These epigenetic clocks have been shown to predict morbidity and mortality in population cohorts (Fransquet et al. 2019; Oblak et al. 2021; Chervova et al. 2024; Crimmins et al. 2025). When the statistical models predicting morbidity or mortality are adjusted with common risk factors of non‐communicable chronic diseases, for example BMI, smoking, alcohol consumption or exercise, the associations between epigenetic clocks and future health outcomes are usually retained, though they can be attenuated (Belsky et al. 2022; Lu et al. 2019; Hillary et al. 2020). Notably, these risk factors are also good predictors of morbidity and mortality on their own (Zhang et al. 2021; Barbaresko et al. 2018; Loef and Walach 2012; Chudasama et al. 2020). Unsurprisingly, these various risk factors associated with increased morbidity and mortality are also associated with increased epigenetic age (Wang et al. 2023; Nannini et al. 2023; Fox et al. 2023; Quach et al. 2017; Chervova et al. 2024; Oblak et al. 2021; Faul et al. 2023; McCarthy et al. 2023). As the above‐mentioned risk factors are easy and affordable to measure, especially in comparison to DNA methylation, it is important to determine whether epigenetic clocks outperform them or provide additional predictive value by capturing aspects of biological ageing not reflected by these simple risk factors. This is especially crucial if the epigenetic clocks are to be used in clinical settings or as tools of personal health monitoring.

Here, we have compared the predictive value of various epigenetic clocks and traditional risk factors of non‐communicable chronic diseases in predicting disease incidence in a 7‐to‐9‐year follow‐up in the middle‐aged Young Finns Study cohort (YFS, Pahkala et al. 2026). At baseline, the participants were aged 34–49 years and were free from studied non‐communicable chronic diseases, such as cardiometabolic diseases, hypertension, cancer and steatotic liver disease (for a detailed list of conditions included, see Tables S1 and S2). Of the 1108 individuals included in the analysis, 222 (20.0%) were diagnosed with one or more non‐communicable chronic disease or condition during the 7‐to‐9‐year follow‐up. Subjects with incident disease during the follow‐up were older and had a higher BMI and waist‐to‐hip‐ratio at baseline. The epigenetic clocks included in the analyses were age deviation (AgeDev) for Hannum (Hannum et al. 2013), Horvath (Horvath 2013), PhenoAge (Levine et al. 2018) and GrimAge (Lu et al. 2019) and their principal component (PC) derivatives (Higgins‐Chen et al. 2022), as well as DunedinPACE (Belsky et al. 2022). For detailed description of methodology, see Supplementary Methods in Supporting Information.

In a minimally adjusted model (age, sex and Illumina array version), _PC_PhenoAge_AgeDev_, GrimAge_AgeDev_ and DunedinPACE were statistically significant predictors of non‐communicable disease incidence during the 7‐to‐9‐year follow‐up (Table 1). These results are in line with published literature, as second‐ and third‐generation clocks have been shown to be better predictors of future health outcomes as compared to first‐generation clocks (Levine et al. 2018; Lu et al. 2019; Belsky et al. 2022).

**TABLE 1:** Association between epigenetic clocks and incidence of any ageing‐associated non‐communicable chronic disease or condition (cardiometabolic diseases, hypertension, cancer, steatotic liver disease, Table S2) in a 7‐to‐9‐year follow‐up (*n* = 1108).

	OR (95% CI)	*p*
Horvath_AgeDev_	1.00 (0.86–1.16)	0.995
_PC_Horvath_AgeDev_	1.11 (0.95–1.29)	0.181
Hannum_AgeDev_	1.08 (0.93–1.25)	0.338
_PC_Hannum_AgeDev_	1.07 (0.92–1.25)	0.352
PhenoAge_AgeDev_	1.15 (0.99–1.34)	0.066
_PC_PhenoAge_AgeDev_	1.18 (1.01–1.37)	**0.032**
GrimAge_AgeDev_	1.20 (1.03–1.39)	**0.015**
_PC_GrimAge_AgeDev_	1.13 (0.98–1.31)	0.094
DunedinPACE	1.19 (1.02–1.39)	**0.024**

*Note:* Logistic regression models were adjusted for age, sex and Illumina array version. Statistically significant *p*‐values are presented in bold.

Abbreviations: 95% CI, 95% confidence interval; OR, odds ratio.

Next, we adjusted the regression models with easy and affordable to measure risk factors of common chronic diseases, namely smoking, alcohol consumption, waist‐to‐hip‐ratio (WHR), and BMI. In these fully adjusted models, none of the analysed epigenetic clocks were statistically significant predictors of disease incidence during follow‐up (Table S3). The attenuation was mainly due to anthropometric variables, as in a model adjusted only for lifestyle variables (smoking and alcohol consumption), GrimAge_AgeDev_ and DunedinPACE remained statistically significant predictors of disease incidence during follow‐up (Table S4).

We then compared a model consisting of the traditional risk factors (age, sex, smoking, alcohol consumption, WHR and BMI) to the minimally adjusted epigenetic clock models in relation to prediction of incidence of non‐communicable diseases during the 7‐to‐9‐year follow‐up. The model consisting only of the traditional risk factors showed better discriminative performance as compared to any of the models containing the epigenetic clocks (Figure 1). However, none of the statistical models show particularly good discriminative performance. The differences in AUC between the traditional risk factor model and minimally adjusted epigenetic clock models were statistically significant (DeLong test *p*‐value < 0.05, Table S5).

**FIGURE 1:**
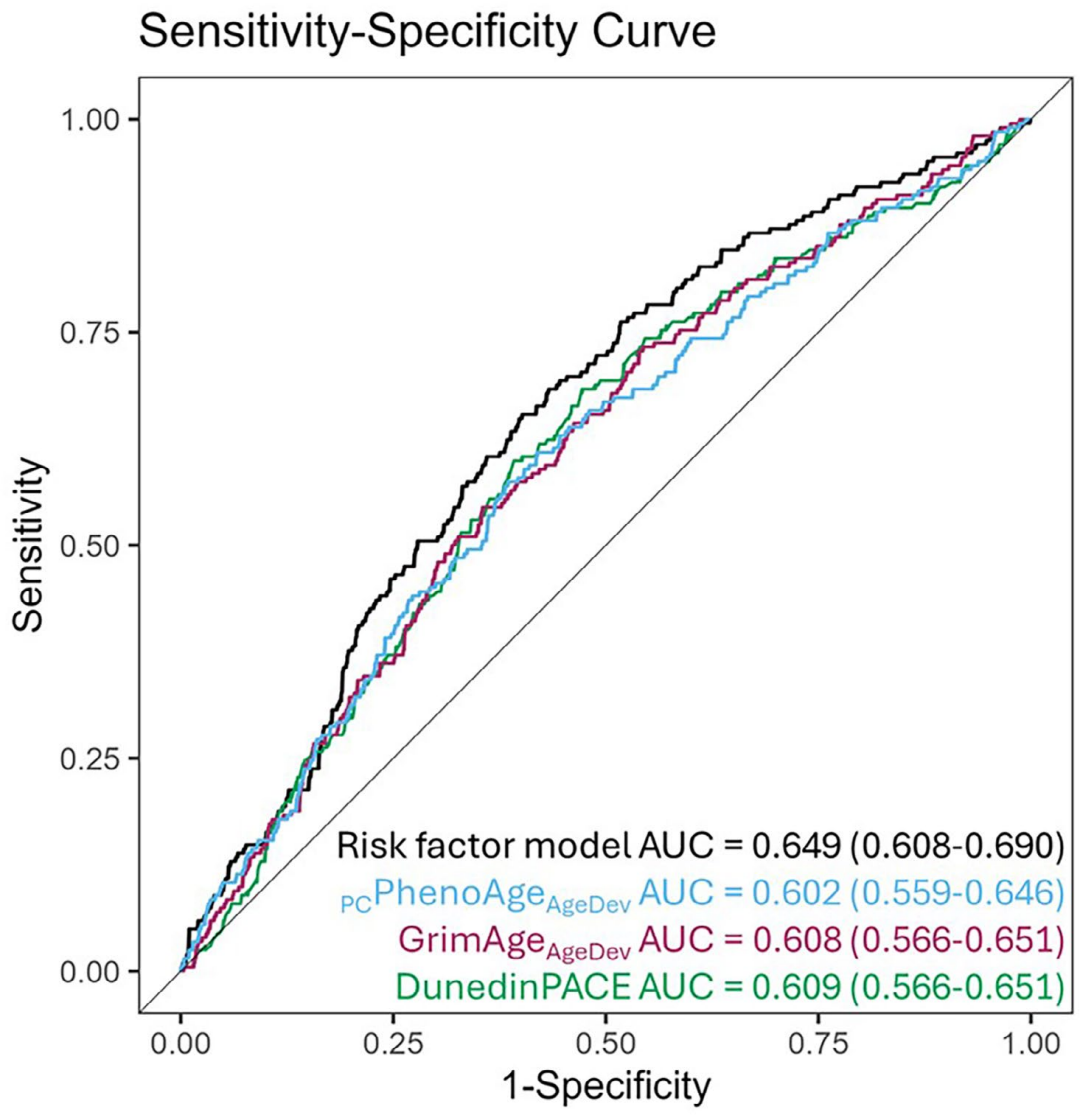
Receiver operating characteristics (ROC) curves comparing the discrimination performance of regression models consisting of traditional risk factors (risk factor model; age, sex, WHR, BMI, smoking, alcohol consumption) and minimally adjusted (age, sex, Illumina array) epigenetic clock models in relation to incidence of non‐communicable disease during a 7‐to‐9‐year follow‐up.

In previous studies, adjusting for traditional risk factors has been shown to attenuate and even eliminate (Föhr et al. 2021) the association between epigenetic clocks and future health outcomes (Belsky et al. 2022; Lu et al. 2019; Hillary et al. 2020). Our finding that risk factors of common chronic disease outperform epigenetic clocks and the previously reported attenuation suggests that epigenetic clocks and traditional risk factors capture overlapping information, raising the question of whether these clocks contribute unique predictive value beyond traditional risk factors. In our study, individuals who remained healthy during follow‐up were younger and had lower WHR and BMI at baseline, further indicating that the simple risk factors are meaningful predictors of morbidity (Zhang et al. 2021; Barbaresko et al. 2018).

The unique contribution of an epigenetic clock beyond established risk factors can be formally assessed using nested model comparisons, which test whether inclusion of the clock significantly improves model fit by adding information beyond that captured by traditional risk factors (Cook 2018). In our study, such analyses were not feasible, as none of the studied clocks were statistically significant predictors of morbidity in fully adjusted models (Vickers et al. 2011). Notably, in studies where epigenetic clocks remain statistically significant predictors of future health outcomes after adjustments with traditional risk factors, formal assessments of their added predictive value have typically not been presented.

Compared to previous studies, where epigenetic clocks retained statistical significance also when adjusted for common risk factors, our cohort is younger and of a narrower age range, which could contribute to the poorer performance of epigenetic clocks in our cohort. In addition, our study is limited by a modest sample size. Further studies in varying population cohorts, especially of varying age ranges, are needed to establish the demographic in which the epigenetic clocks would have the highest predictive performance and where they would provide the most added value. Especially interesting would be to establish whether the epigenetic clocks are predictive of future health outcomes in populations where the traditional risk factors are less informative, for example in secondary prevention, or in populations where there is less variation in the traditional risk factors, for example among individuals strictly adhering to healthy lifestyles.

As epigenetic clock measurements are already sold to consumers as tools of personal health monitoring, it should be shown they provide meaningful information also in the demographic most likely to purchase them. To the best of our knowledge, there is no data available on who are most likely to have their epigenetic age measured, but for other self‐monitoring solutions, wearable devices, it is known that those who use them in comparison to those who do not are younger, healthier and more educated (Dhingra et al. 2023).

Epigenetic clocks potentially capture multiple aspects of the biological ageing process, and therefore when studying ageing itself or the molecular mechanisms of ageing, it is a more appropriate metric than a simple disease risk factor. However, if the aim is to predict future health outcomes for individuals, a simpler approach can be the cost‐effective one. Given the higher cost of DNA methylation measurements, the added value of epigenetic clock measurements over traditional risk factors should be considerable to justify their use.

## Author Contributions

Saara Marttila planned the study. Saara Marttila and Daria Kostiniuk designed the data analysis. Daria Kostiniuk and Flóra Székely performed the data analysis. Leo‐Pekka Lyytikäinen, Jo Ciantar, Sonja Rajić, Pashupati P. Mishra, Terho Lehtimäki, Katja Pahkala, Suvi Rovio, Juha Mykkänen and Olli Raitakari provided data. Terho Lehtimäki, Olli Raitakari, Emma Raitoharju and Saara Marttila acquired funding. Saara Marttila wrote the first draft of the manuscript, Daria Kostiniuk participated in writing the first draft of the manuscript and all co‐authors read and revised the final manuscript.

## Funding

The Young Finns Study has been financially supported by the following organisations: the Academy of Finland (grants 356405, 322098, 286284, 134309 (Eye), 126925, 121584, 124282, 129378 [Salve], 117797 [Gendi] and 141071 [Skidi]); the Social Insurance Institution of Finland; Competitive State Research Financing of the Expert Responsibility area of Kuopio, Tampere and Turku University Hospitals (grant X51001); the Juho Vainio Foundation; the Paavo Nurmi Foundation; the Finnish Foundation for Cardiovascular Research; the Finnish Cultural Foundation; the Sigrid Juselius Foundation; the Tampere Tuberculosis Foundation; the Emil Aaltonen Foundation; the Yrjö Jahnsson Foundation; the Signe and Ane Gyllenberg Foundation; the Diabetes Research Foundation of the Finnish Diabetes Association; EU Horizon 2020 (grant 755320 for TAXINOMISIS and grant 848146 for To Aition); the European Research Council (grant 742927 for MULTIEPIGEN project); the Tampere University Hospital Supporting Foundation; the Finnish Society of Clinical Chemistry; the Cancer Foundation Finland; pBETTER4U_EU (Preventing obesity through Biologically and bEhaviorally Tailored inTERventions for you; project number: 101080117); CVDLink (EU grant nro. 101137278); and the Jane and Aatos Erkko Foundation. Pashupati P. Mishra (grant no. 349708) and Emma Raitoharju (grants 330809 and 338395) were supported by the Academy of Finland.

Researchers in the current study have been supported by Yrjö Jahnsson Foundation, Juho Vainio Foundation, Päivikki and Sakari Sohlberg Foundation, Paulo Foundation, The Finnish Foundation for Cardiovascular Research and Pirkanmaa Regional Fund of the Finnish Cultural Foundation.

## Conflicts of Interest

The authors declare no conflicts of interest.

## Supporting information


**Table S1:** Demographics of the study population at baseline (*n* = 1108). At baseline, the study subjects did not have any of the conditions or medications listed in Table S2.
**Table S2:** Incident non‐communicable disease frequencies at follow‐up. One individual could gain more than one condition; therefore, the sum of frequencies exceeds the total number of individuals who gained a condition during follow‐up (*n* = 222).
**Table S3:** Association between epigenetic clocks and incidence of any ageing‐associated non‐communicable chronic disease or condition (cardiometabolic diseases, hypertension, cancer, steatotic liver disease, for detailed list see Table S2) in a 7‐to‐9‐year follow‐up (*n* = 1039) in the fully adjusted logistic regression model (age, sex, Illumina array version, smoking, alcohol consumption, WHR, BMI). OR; odds ratio, 95% CI; 95% confidence interval.
**Table S4:** Association between epigenetic clocks and incidence of any ageing‐associated non‐communicable chronic disease or condition (cardiometabolic diseases, hypertension, cancer, steatotic liver disease, for detailed list see Table S2) in a 7‐to‐9‐year follow‐up (*n* = 1039) in serially adjusted logistic regression models. All models were adjusted for Illumina array version in addition to phenotypes given in the column titles. OR; odds ratio, 95% CI; 95% confidence interval.
**Table S5:** Comparison of the discrimination performance between minimally adjusted (age, sex, Illumina array) epigenetic clock models and a model composed of easy and affordable to measure risk factors (age, sex, smoking, alcohol consumption, WHR, BMI) models in relation to incidence of non‐communicable disease during a 7‐to‐9‐year follow‐up. Higher AUC values indicate better discriminatory performance. Differences in AUC between models were assessed with DeLong test. For the ROC curves, see Figure 1.
**Table S6:** Correlation between chronological age and the different epigenetic clocks used in the study (*n* = 1108). Note, that while age deviation (AgeDev) values were used in regression analyses presented in the main body and in the Supporting Information, these correlations are presented for the epigenetic clock values themselves (representing epigenetic age in years).

## Data Availability

The datasets generated and/or analysed during the current study are not publicly available due to restrictions imposed by Finnish legislation but are available from the corresponding author/data sharing committee upon a reasonable request.
